# Cultivation of Entrepreneurial Talents Through Virtual Entrepreneurship Practice in Higher Education Institutions

**DOI:** 10.3389/fpsyg.2021.690692

**Published:** 2021-07-29

**Authors:** Hengjie An, Yuanyuan Xu

**Affiliations:** ^1^Shen Junru Law School, Hangzhou Normal University, Hangzhou, China; ^2^School of Law, Zhejiang Gongshang University, Hangzhou, China

**Keywords:** higher education institution, virtual entrepreneurship education, entrepreneurial talents, talent training mode, entrepreneurial knowledge structure

## Abstract

The purpose is to study the influence of virtual entrepreneurship practice on entrepreneurial talent cultivation and innovation and entrepreneurship education in higher education institutions. First, a questionnaire is designed from three aspects: entrepreneurial awareness, entrepreneurial psychological quality, and entrepreneurial knowledge structure. Afterward, the questionnaire is issued to 200 students from a college in Shaanxi province, and SPSS 25 is chosen to analyze and characterize the virtual entrepreneurship practice education and the innovation and entrepreneurship education in higher education institutions. The results show that among all the subjects, nearly 20% of them do not understand entrepreneurship, nearly 85% of them concern about the risk of entrepreneurship, and nearly 70% of them take a negative attitude toward entrepreneurial failure. Meanwhile, the subjects have not formed an independent view on entrepreneurship, the subjects’ basic entrepreneurial quality is poor, their entrepreneurship awareness is weak, and their entrepreneurial psychological quality is poor. The subjects lack entrepreneurial quality rather than entrepreneurial knowledge. Finally, some suggestions are put forward on education on virtual entrepreneurship practice in higher education institutions from four aspects: improving the external environment of entrepreneurship, improving the entrepreneurship curriculum in higher education institutions, improving the teaching staff, and developing the practice bases for entrepreneurship education. The results provide some ideas about promoting the comprehensive reform of talent training mode in higher education institutions.

## Introduction

The 21st century has witnessed the appearance and development of the knowledge economy, which is based on knowledge, led by high technology, and driven by innovation. With the advent of the era of the knowledge economy, the traditional economic development model has been impacted, and the requirements for talents are getting higher and higher. Especially, people with an outstanding entrepreneurial spirit are in higher demand in economic development. Lai and Yu (2021) pointed out that entrepreneurship education should be instructed regularly to train well-qualified entrepreneurial talents. Under a market economy, people with an entrepreneurial spirit can promote economic development and scientific and technological progress (Jin and Chen, 2020). Innovation is the source of economic development and scientific and technological progress. Therefore, more scientific and up-to-date practical teaching modes should be adopted by higher education institutions to continuously improve the overall human resource quality, cultivate entrepreneurial talents, tailor to the needs of the market economy development, and finally, improve the comprehensive national strength of China (Chenghao et al., 2019). Besides, entrepreneurship education is a crucial part of quality education in China. Thus, the research on entrepreneurship education and entrepreneurship-oriented personnel training can provide a reference for the theoretical content of entrepreneurship education in China and help broaden the research field of higher education institutions.

Here, it is believed that Maker Education praises commercialization highly. Meanwhile, the Internet of things (IoT) and Artificial Intelligence (AI) technology can be applied to construct Maker space in higher education institutions. Then, digital learning resources, teachers, students, and designers can be gathered, coordinated, and shared through the Make space to construct a new paradigm, namely, the online-offline Maker space. Therefore, higher education institutions should integrate the Maker space, carry out innovation and entrepreneurship competition and incubation projects, and study the integrated talent cultivation mode of Maker Education and entrepreneurship education, thus improving students’ innovation and entrepreneurship ability.

The cultivation of entrepreneurial talents and entrepreneurship education in Chinese higher education institutions are still in their infancy. Regarding the modes of entrepreneurship education, researchers have put forward diverse ideas while have not formed a universal and perfect system or theory. The earliest foreign entrepreneurship education has developed in the United States. Two representative entrepreneurship education modes have been developed after decades of development, which are focalization mode and university-wide entrepreneurship education mode. With just over a decade of development, some practical experiences have also been accumulated in Chinese higher education institutions. Regarding teaching content, representative entrepreneurship education includes the classroom-centered and quality-oriented entrepreneurship education mode, the knowledge and skill-oriented entrepreneurship mode, and the mixed-mode entrepreneurship education (Chugh and Ruhi, 2018).

In China, the theoretical research of entrepreneurship education and the virtual entrepreneurship practice is just beginning. Most researchers focus on the cultivation of entrepreneurial talents. There is not much research on entrepreneurial talent cultivation through virtual entrepreneurship practice. The innovation point is to study the cultivation of entrepreneurial talents in higher education institutions through the virtual entrepreneurship practice and establish the corresponding evaluation standards for students’ entrepreneurial quality. Consequently, the entrepreneurial quality index system is established under the virtual entrepreneurship practice mode. The purpose is to mine the problems existing on the virtual entrepreneurship of higher education institutions under commercialization, and discover relevant issues in students’ entrepreneurial awareness and entrepreneurial quality through the questionnaire, and put forward some improvement plans according to analysis (Liu et al., 2021).

## Related Work

Many researchers have made contributions to the research of entrepreneurship-oriented personnel training. Yan (2018) pointed out that the essence of personalized education is innovation education that respects individual differences. With the continuous development of the times, the ability of innovation and entrepreneurial ability has become the key to reflect the comprehensive national strength. In response to the call of construction of an innovative country in China, students’ personalized thinking and problem-solving abilities should be cultivated, and their creativity and subjectivity should be respected, laying a solid foundation for students’ innovation and entrepreneurship in the future. Here, the current situation of domestic and international personalized education is analyzed to promote the development of students, and the existing problems and solutions are pointed out. The research aims to accelerate innovation-driven social development in China. This will lay the foundation for further improvement on the teaching quality of art courses for education majors. Rakhmatov and Nizamov (2020) pointed out that the combination of innovation and entrepreneurship education and vocational education is a crucial measure to promote high-quality employment and entrepreneurship for graduates. The problems of teaching innovation and entrepreneurship through computer technology were analyzed, and the reform mode based on the four wheels (four dimensions of economic growth, including human resource development, natural resource development, capital formation, and technological progress) was put forward. Two innovation and entrepreneurship educational concepts were formed through the integration and optimization of professional talent training programs: (1) Various forms of school–enterprise cooperation platforms should be established. (2) A reasonable management and incentive mechanism should be established to realize the dynamic integration of teachers’ and students’ education and innovation (Wang, 2020). Ong et al. (2018) pointed out that the problems of innovation and entrepreneurship management and the economic incentive mechanism are of great significance. Li and Jiao (2021) pointed out that the IoT technology and big data promote the innovation and transformation of all walks of life. Higher education institutions were the main talent incubation bases, and educational reform was continuously being explored. Particularly, the professional course structure was being upgraded and transformed through the integration of information technology with the traditional education model. First, the necessity was analyzed for college students’ innovation and entrepreneurship education under Internet+. Then, the status of innovation and entrepreneurship education in higher education institutions was investigated through questionnaire on universities of Shandong province. Finally, some suggestions were given to promote the development of innovation and entrepreneurship education and practice. Yang (2020) studied the influence of the positive entrepreneurial quality on innovation and entrepreneurship education, as well as the development of creative education in higher education institutions. The innovation and entrepreneurship education and the creative education in higher education institutions were analyzed and characterized through a questionnaire designed based on *Chinese College Students’ Positive Psychological Characteristics Scale* and SPS. The results show that liberal arts, science, and engineering students’ positive entrepreneurship psychology are affected by different factors. The family economy exerts the most obvious influence on liberal arts students, sports activities exert the most obvious impact on science students, while the influence of grades on engineering students is the most obvious. The average score of innovation and entrepreneurship ability of college students is about 3.0 points, indicating that the overall innovation and entrepreneurial ability of college students is general. Besides, there are differences in the development of the creative education model in the East and West. Generally, positive entrepreneurial psychology plays a positive role in cultivating students’ healthy entrepreneurial quality and promoting the development and practice of maker education. Ruihua et al. (2021) analyzed the status of innovation and entrepreneurship education mode in higher institutions and put forward the innovation strategy for the existing problems. To sum up, globally, there are various kinds of research on innovation and entrepreneurship education, university Maker education mode, and psychological innovation and entrepreneurship education, while these kinds of research are just picking up in China. Moreover, the research on Maker education mode under the perspective of positive psychology is rare. Hence, the role of Maker education in innovation and entrepreneurship education is explored based on positive psychology (Hayes and Wynyard, 2018).

## Basic Theories of Entrepreneurial Quality and Entrepreneurship Education

### Commercialization Philosophy

Many policies have been issued to encourage students’ entrepreneurship since the development of entrepreneurship education and college students’ entrepreneurship activities in China. Supported by the national macro policy, social environment, and higher education institutions, the National College Students’ Entrepreneurship plan competition, and College Students’ Excellent Team Entrepreneurship competition become increasingly popular (Soegoto and Luckyardi, 2020). Wu and Wu (2017) systematically reviewed the relevant literature on entrepreneurship education in the Asia pacific region in the past decade, summarized and analyzed the previous research results on emotional expression, and finally, commented on the key issues. The purpose of the competition is to cultivate students’ entrepreneurial concepts and awareness and improve their entrepreneurial ability. Although entrepreneurship education has developed rapidly in China in recent years, most of the entrepreneurship activities are only carried out among several universities, only a few students participate in entrepreneurship activities in schools, and only a few entrepreneurial achievements have been commercialized. In China, several representative entrepreneurial models have been proposed, and commercialization approaches have been explored. The best entrepreneurial model is characterized by commercialized operation, the establishment of an entrepreneurial park, teaching students how to start a business, and providing financial resources and services for students. However, these entrepreneurial models are not always successful in commercialization because of limited scale and minor social influences. The crux lies in that entrepreneurship education is not closely related to commercialization. Entrepreneurial activities should be normalized to successfully commercialize the entrepreneurial achievements of college students (Bischoff et al., 2018,2021).

**FIGURE 1:**
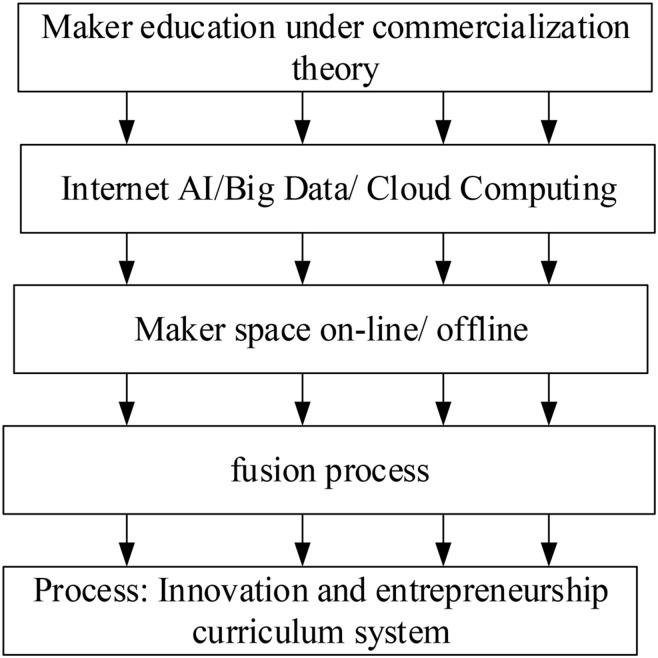
The cultivation path of entrepreneurial talents in Maker Education under commercialization theory.

The existing Maker space in higher education institutions is an open resource entrepreneurial practice platform with a commercialization concept. Maker space provides a new understanding for the cultivation of entrepreneurial and innovative talents in higher education institutions. In higher education institutions, the Maker space provides a commercialized practice environment for ordinary students. The integration of online-offline Maker space projects and commercialized projects help cultivate students into high-quality talents with both professional knowledge and innovation and entrepreneurial ability. Figure 1 displays the cultivation path of entrepreneurial talents under the business theory of Maker education.

### Theories of Entrepreneurship

The words entrepreneurship or enterprise both have the meaning of setting up a business. The modern concept of entrepreneurship is derived from the entrepreneur. Therefore, entrepreneurship and entrepreneurs are closely related, and the meaning of entrepreneurship becomes very rich. The connotation of entrepreneurship varies greatly from the simplest nature to the complex process (Setiyadi and Setiawan, 2020; Xiao and Chen, 2020). Most scholars define entrepreneurship from the perspective of economics. For example, Churchill, the famous British Prime Minister, agrees with the author of entrepreneurship art that entrepreneurship is an opportunity that can create value through innovation without considering capital limits, human resource restriction, and environmental restriction (Sudarwati et al., 2020). In China, some researchers have pointed out that entrepreneurship is a process, where opportunities need to be discovered and captured, and new products or services are created and the value of products or services can be embodied. Hence, the essence of entrepreneurship can be defined as the idea and behavior of human beings, and it can be categorized into special entrepreneurship and general entrepreneurship. Meanwhile, innovation is the core of entrepreneurship in both a narrow sense and a broad sense. Entrepreneurship refers to the spirit that entrepreneurs manifest when seeking and grasping opportunities, integrating resources, and creating value under an unpredicted economic environment. Particularly, independent entrepreneurship of college students refers to the process of creating new jobs in various ways, such as technology equity investment and self-financing (Chenghao et al., 2019; Svensson and Bogren, 2019). In the late 1980s, entrepreneurship education has been first proposed by western countries and United Nations Educational, Scientific, and Cultural Organization (UNESCO). In a broad sense, entrepreneurship education can cultivate the most innovative person and can get employees paid more. Entrepreneurship personnel training can be divided into two dimensions. The first dimension is to cultivate entrepreneurs who can turn knowledge and technology into business opportunities. The second dimension is to cultivate talents with technical, social, and management skills. This group of people has a pioneering, adventurous, and entrepreneurial spirit, as well as independent working ability among the non-entrepreneurs (Bo, 2017; Marniati and Wibawa, 2020).

**FIGURE 2:**
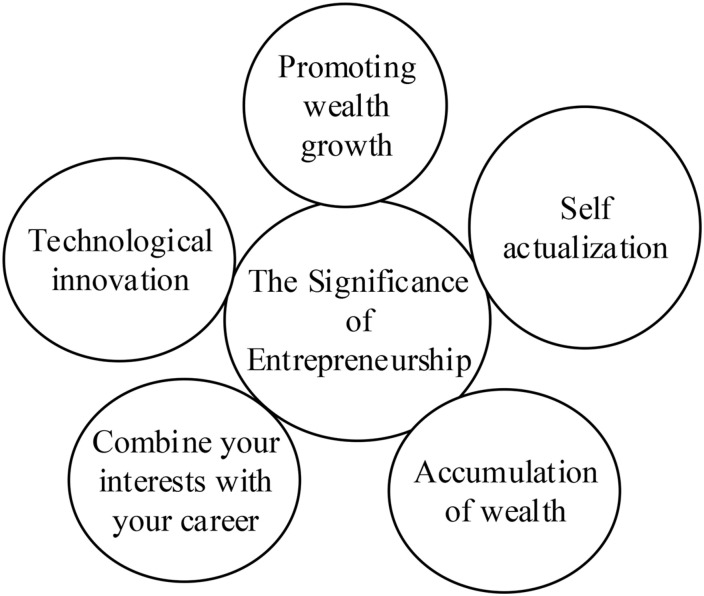
The significance of entrepreneurship.

The second kind of talents are, in today’s business environment, being paid more and more attention to and are demanded by most ventures. The typical entrepreneurship education includes the Master of Business Administration (MBA) education, which is tailored for people at work, and there is popular entrepreneurship education for students in higher education institutions. Entrepreneurship education in school can develop and improve the basic quality of entrepreneurship of students and strengthen their survival skills, competitiveness, and entrepreneurship, thus providing composite talents for society (Davies, 2019; Jay and Jones, 2019). Nowadays, general entrepreneurship education has attracted more and more attention. Compared with the special entrepreneurship-the literal meaning of the word *entrepreneurship*, general entrepreneurship has broader meanings. Derived from general entrepreneurship, entrepreneurship education has revolutionized the traditional education model which focuses on specific operation skills. Instead, more attention has been paid to innovation ability and comprehensive quality (Ferro, 2020). Wu et al. (2018) pointed out that although entrepreneurship education involved many teaching activities, traditional classroom lectures, case studies, and group discussions were the most common methods, while information and communication technologies could better convey students’ business philosophy. Therefore, entrepreneurship education can provide a new perspective and platform for the growth and comprehensive quality improvement of college students. Figure 2 illustrates the significance of entrepreneurship from five aspects.

**FIGURE 3:**
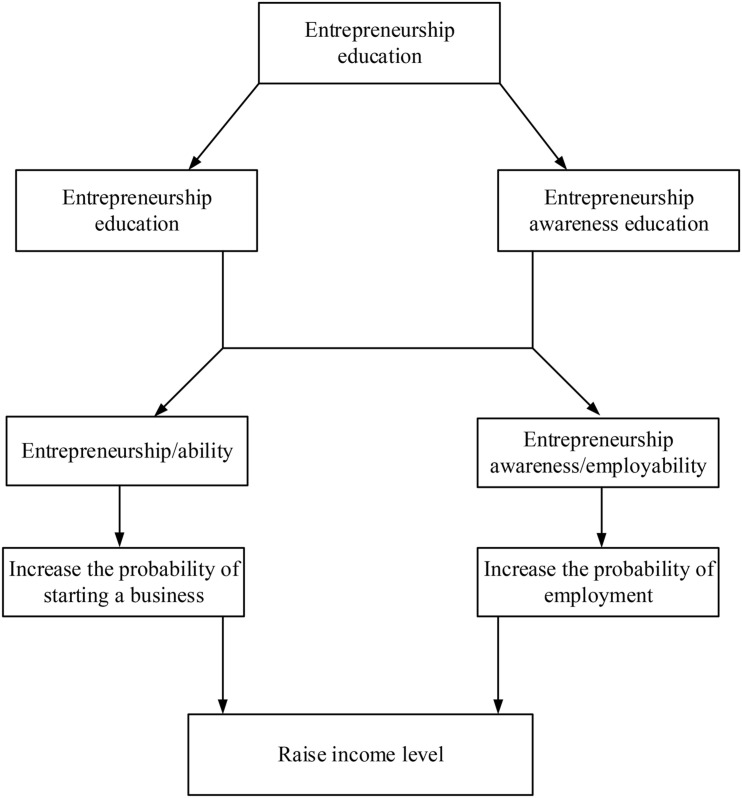
Improvement mechanism of entrepreneurship education.

There is still no clear definition of talent training mode except for some researchers’ descriptions. Some have described talent training mode as the specification and methods for personnel training. The basic characteristics of talents in higher education institutions are determined by the talent training mode (Gomezreino, 2018; Hayes and Wynyard, 2018). Others have explained the talent training mode from the operational level and point out that talent training mode refers to a structure that the school establishes for students, including knowledge, abilities, and qualities, as well as the realization of the structure. Specifically, talent training modes include the training objectives, training specifications, and basic training methods of talents. Based on the definition of talent training mode, here, the talent training mode can be analyzed from four dimensions. (1) The talent training mode is the concretization of a specific talent training thought. (2) Talent training mode is a complex multi-factor dynamic process and a comprehensive system to train talent. (3) Talent training mode is a replicable framework of talent training activities. (4) Each talent training mode has its unique objects, and there is no universal talent training mode. Figure 3 displays the improvement mechanism of entrepreneurship education (Caroline and Halimat, 2018; Macaro et al., 2018).

### The Theory and the Quality Indices of Entrepreneurial Talents

**FIGURE 4:**
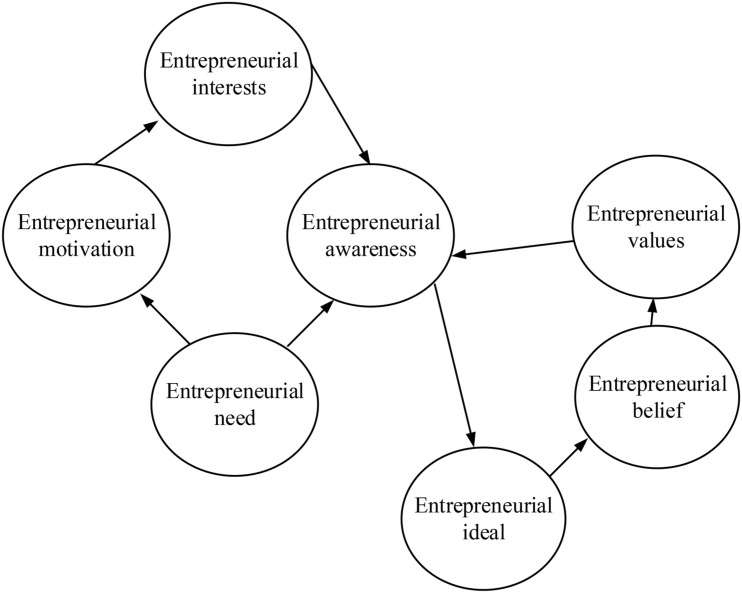
The logic relationship among the elements of entrepreneurial awareness.

Entrepreneurship education is the foundation and core of the cultivation of entrepreneurial talents. Based on the above analysis, entrepreneurial talents can be divided into two categories. The first category refers to the college students who receive entrepreneurship education in school and exert specialties in the work after graduation. The second category refers to the job-creators. Compared with the first category, job-creators can provide jobs for others to promote social development. Scholars have defined the concept of entrepreneurial talents from different perspectives (Kupfer, 2018; Macaro et al., 2018). At first, some scholars have defined entrepreneurial talents as those who are willing to take risks and have innovative thinking and entrepreneurial awareness from the perspective of psychology (Tran et al., 2018; Zavale, 2018). With in-depth research, the definition of entrepreneurial talents has been changed. Some researchers define entrepreneurial talent as a compound talent with an entrepreneurial spirit and high comprehensive quality. The cultivation of entrepreneurial talents can be divided into two dimensions. The first dimension cultivates entrepreneurs who can transform knowledge and technology into business opportunities. The second dimension cultivates people with venturous spirit and entrepreneurial skills among non-entrepreneurial people. Thus, entrepreneurial talents refer to compound high-quality talents with a strong entrepreneurial spirit, entrepreneurial awareness (Hu et al., 2020), abundant professional and entrepreneurial knowledge, and profound humanistic quality. Figure 4 shows the description of the logical relationship among the elements of entrepreneurial awareness.

In Figure 4, entrepreneurial awareness is a deeply rooted internal need of entrepreneurs, namely, entrepreneurial need or entrepreneurial intention, which is the motivation of the very first entrepreneurial activities and the basic form of all the elements in entrepreneurial awareness. Driven by psychological motives, entrepreneurial need transforms into entrepreneurial motivation. The entrepreneurial need is the base of entrepreneurial motivation, and it is manifested through entrepreneurial motivation which can transform into entrepreneurial interest. Entrepreneurial interest stimulates the entrepreneurial emotion of entrepreneurs, so that entrepreneurial awareness becomes entrepreneurial ideal. The entrepreneurial ideal is the advanced form of entrepreneurial awareness, which is pursuit by most entrepreneurs. Therefore, the ultimate purpose of entrepreneurial awareness cultivation is to develop the entrepreneurial ideal. Entrepreneurial beliefs are formed during entrepreneurial practices and are the spiritual support for entrepreneurial activities and the entrepreneurial ideal. The logical relationship of a series of entrepreneurial beliefs forms entrepreneurial values.

Table 1 is the quality index system of entrepreneurial talents.

**TABLE 1:** Quality index system of entrepreneurial talents.

EA		ES		
Entrepreneurial intention/Motive	Entrepreneurial values	Innovation	Venturous	pragmatic

**EQ**		**E**		

Daring	Independence	Organization/communication	Learning/management	Judgment/decision-making

In Table 1, EA denotes entrepreneurial awareness, ES means entrepreneurial spirit, EQ stands for entrepreneurial quality, and E represents entrepreneurship ability.

### Questionnaire Design for Entrepreneurial Quality of College Students

Here, the research subjects are selected from the students of college A in Shaanxi Province, which has implemented entrepreneurship education for a while. According to the number of students in college A, 200 questionnaires are issued, and 196 valid questionnaires are recovered, with an effective rate of 98%. The subjects’ basic information and answers of the valid questionnaires are collected and statistically analyzed through SPSS 25. Table 2 displays the statistical information of the subjects. In the sample, there are 98 male college students and 98 female students, accounting for 50 and 50%, respectively. The proportion of freshmen, sophomores, juniors, and seniors is 25%, respectively. The subjects’ other information, such as grade and gender, are basically in line with the basic situation of college A, as well as the actual situation of higher education institutions in Shaanxi Province. Thus, the questionnaire is effective. Table 2 presents the basic information of the subjects. Specifically, questionnaires are distributed to students during class hours and collected with the assistance of the teachers on the spot. After all the questionnaires are collected, the invalid questionnaires are removed, and effective questionnaires are statistically analyzed. Table 3 presents the questionnaire analysis of EA.

**TABLE 2:** Basic information of subjects.

	Variable	Number	percentage
Gender	Male	98	50
	Female	98	50
	Freshman	54	25
Grade	Sohpormore	54	25
	Junior	54	25
	Senior	54	25

**TABLE 3:** The EA questionnaire.

Question 1 What is entrepreneurship?			Question 2 What is your attitude toward college students’ entrepreneurship?		
A. Start a company	B. Start a career at will	C. Other	A. Favorable	B. Unfavorable	C. Neutral

**Question 3 Are there any entrepreneurial intentions?**			**Question 4 Why do you plan to start a business?**		

A. Ready to start a business	B. Maybe ready	C. Never have considered it	A. No appropriate job has been found	B. Want more money	

The entrepreneurial psychological quality questionnaire is shown in Table 4.

**TABLE 4:** The entrepreneurial psychological quality questionnaire.

Question 1 What is your choice against the entrepreneurial risk?			Question 2 How do you make your plan?		
A. Without risk concerns invest alone	B. Partnership investment and shared risk	C. Under-bearing capacity	A. Imitate others	B. Plan for yourself and listen to others	C. Discuss with others before making a decision

**Question 3 How do you feel when you choose to start a business?**			**Question 4 What will you do after entrepreneurial failure?**		

A. Confident	B. Confused	C. Stressed	A. Abandon	B. Proceed with second pioneering	C. Gain experience and bide their time
					

The questionnaire of college students’ employment values is shown in Table 5.

**TABLE 5:** Employment value questionnaire results.

Question 1 What are factors influencing entrepreneurial choices?				
A. Realization of personal ideals and values	B. High degree of freedom	C. Treatment of high	D. Poor employment situation	E. Influenced by others
**Question 2 What are the essential elements of the entrepreneurial process?**				
A. Record of formal schooling	B. Ability	C. Relationships	D. Others	
**Question 3 What are your solutions to entrepreneurship problems?**				
A. Take a positive attitude	B. Hesitant	C. Give up	D. Let nature takes its course	

## Questionnaire of College Students’ Basic Entrepreneurial Quality

### Sample Reliability and Validity Analysis

Table 6 presents the reliability statistics results.

**TABLE 6:** Reliability statistics.

Cronbach’s Alaph coefficient	Cronbach’s Alaph based on standardized items	Number of terms
0.797	0.766	10

Table 6 illustrates that the Cronbach’s Alpha coefficient can test the reliability of the questionnaire and ensures the consistency of the scoring of each item. The coefficient α is 0.797, indicating that the questionnaire has good reliability and internal consistency.

Here, LISREL8.7 software is chosen for Confirmatory Factor Analysis (CFA), and Table 7 presents the main fitting indexes of the model.

**TABLE 7:** CFA index.

X^2^	df	RMSEA	NNFI	TLI	CFI	IFI
**Fit index**						
2,758.83	1,526	0.067	0.88	0.89	0.91	0.91

Table 7 indicates that the above fitting indexes all meet the conditions of the goodness-of-fit model. The X^2^/df value is 1.8, RMSEA is less than 0.067, and the values of CFI and IFI are greater than 0.9, indicating that the model fitting is good. The fitting indexes all meet the conditions of the goodness-of-fit model, indicating that the factor structure model has good structural validity.

### Actuality of Education on Virtual Entrepreneurship Practice

**FIGURE 5:**
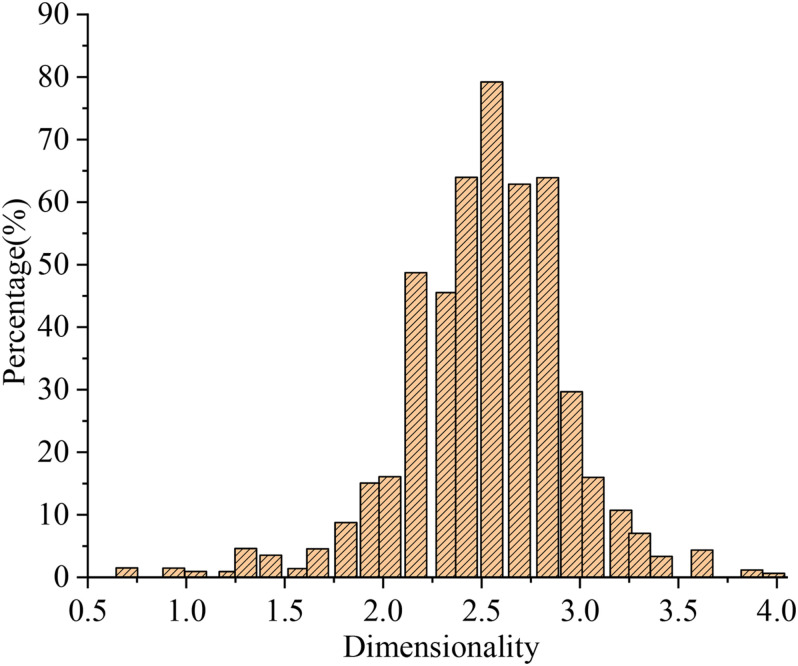
Status of education on virtual entrepreneurship practice.

Figure 5 shows the status of education on virtual entrepreneurship practice in college A.

Figure 5 shows that the status of education on virtual entrepreneurial practice in college A approximates a normal distribution, and the average value is 2.86, almost equal to 3, the theoretical mean of the scale. The results show that virtual entrepreneurship practice education in college A is moderately developed, far from ideal. Meanwhile, the course objective of entrepreneurship practice education have not been met.

Table 8 shows the correlation coefficient between the virtual entrepreneurship practice education and each dimension.

**TABLE 8:** The correlation coefficient between the virtual entrepreneurship practice education and each dimension.

	Dimension	Educational cognition	Curriculum design	Teaching method	Teacher structure
Virtual entrepreneurship practice education	person correlation coefficient	0.631	0.741	0.682	0.624
	Bilateral significance	0.000	0.000	0.000	0.000
	N	516	516	516	516

Table 8 illustrates the correlation coefficient between the virtual entrepreneurship practice education and each dimension. The larger the absolute value of the pearson correlation coefficient is, the stronger their correlation is. Conversely, the smaller the absolute value of the pearson correlation coefficient is, the weaker their correlation is. When 0.8 < | *p*| ≦ 1, the correlation is extremely strong, and when 0.6 < | *p*| ≦ 0.8, the correlation is high. When 0.4 < | *p*| ≦ 0.6, the correlation is moderate, and when 0.2 < | *p*| ≦ 0.4, the correlation is weak. When 0.2 < *p* < 0.2, there is no correlation. The correlation coefficients between each dimension and the virtual entrepreneurship practice education are all above 0.6, indicating that each dimension is positively correlated with the virtual entrepreneurship practice education, and the correlation is significant. Thus, the overall level of each dimension are desirable.

### Questionnaire of EA

**FIGURE 6:**
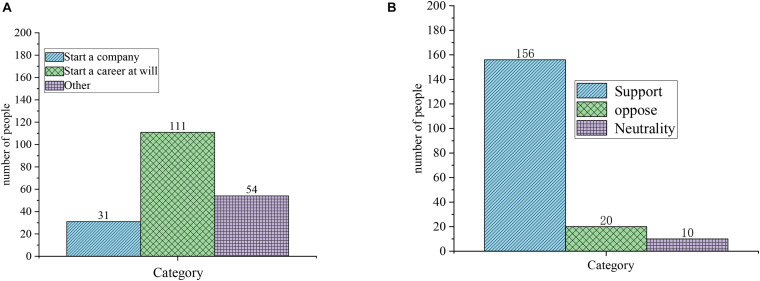
Questionnaire of EA. **(A)** Question 1: What is entrepreneurship. **(B)** Question 2: What is your attitude toward college students’ entrepreneurship.

Figure 6 shows the results of EA questionnaire, and Figure 6A is the result of question 1: what is entrepreneurship? Figure 6B is the result of question 2: What is your attitude toward college students’ entrepreneurship?

**FIGURE 7:**
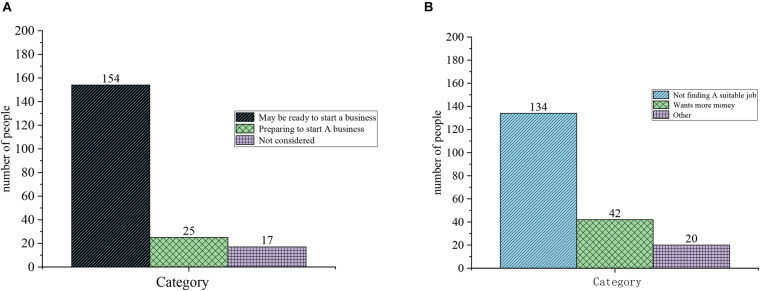
Questionnaire of EA. **(A)** Question 3: Are there any entrepreneurial intentions. **(B)** Question 4: Why do you plan to start a business.

Figure 7 also indicates the results of EA questionnaire, and Figure 7A is the results of question 3: Are there any entrepreneurial intentions? Figure 7B is the result of question 4: Why do you plan to start a business?

Figures 6, 7 illustrate that, currently, college students’ understanding of entrepreneurship is not comprehensive: 31 subjects take the entrepreneurship as the act of starting a company, 111 subjects regard entrepreneurship as starting a career at will, and another 17 subjects hold that they do not have a clear understanding about entrepreneurship. Most subjects favor college students’ entrepreneurship: 156 subjects support college students’ entrepreneurship, while 20 subjects oppose, and 10 subjects are neutral.

For the question of whether they will start a business, only 25 subjects are preparing to start a business, 154 subjects may be ready to start a business, and 17 subjects have not considered starting a business. Many reasons may contribute to this phenomenon, and 134 subjects feel they have no experience and insufficient funds. Therefore, students choose entrepreneurship for many reasons, and nearly half of them choose entrepreneurship because they cannot find suitable jobs. Questionnaire results show that most subjects have expressed positively toward college students’ entrepreneurship. However, on the whole, college students’ entrepreneurial awareness has not been erected, students have not formed an independent understanding of entrepreneurship, and a few of students have misunderstandings about entrepreneurship. Most students are holding a wait-and-see attitude toward entrepreneurship. Few students implement actual entrepreneurial behaviors, and the pursuit of stable work accounts for the majority among students. The linear regression analysis of the questionnaire on “Why do you plan to start a business?” reveals that the significance level P of not finding a suitable job is 0.0001, the significance level P of needing money is 0.029, and the significance level P of other factors is 0.016. Thus, not finding a suitable job has the greatest impact on the cause of college students’ entrepreneurship (Deng et al., 2021).

### Questionnaire of Entrepreneurial Psychological Quality

**FIGURE 8:**
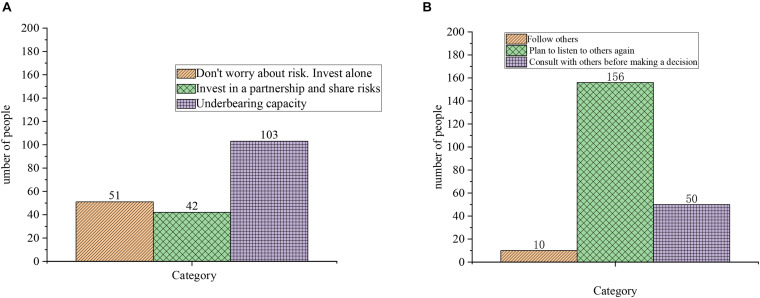
Questionnaire of entrepreneurial psychological quality. **(A)** Question 1: What is your choice against the entrepreneurial risk. **(B)** Question 2: How do you make your plan.

Figure 8 shows the results of the questionnaire on entrepreneurial psychological quality. Figure 8A is the results of Question 1: What is your choice against the entrepreneurial risk? Figure 8B is the result of question 2: How do you make your plan?

**FIGURE 9:**
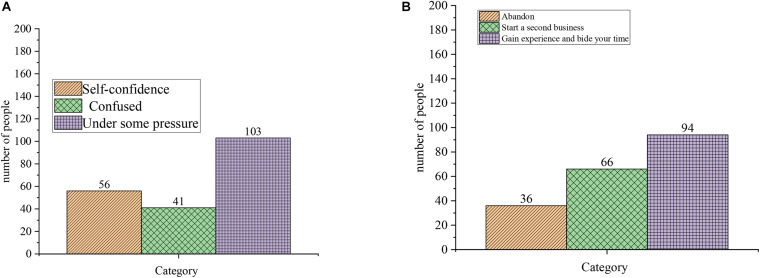
Questionnaire of entrepreneurial psychological quality. **(A)** Question 3: How do you feel when you choose to start a business. **(B)** Question 4: What will you do after entrepreneurial failure.

Figure 9 also demonstrates the results of questionnaire of entrepreneurial psychological quality. Figure 9A is the result of question 3: How do you feel when you choose to start a business? Figure 9B is the result of question 4: What will you do after entrepreneurial failure?

Figures 8, 9 demonstrate that regarding the question about the risk of entrepreneurship, 51 subjects do not worry about the risks and invest alone, 42 subjects choose to invest in a partnership and share risks, and more than half of them think they have an under-bearing capacity. Regarding the question “what you would do when planning,” only 10 students choose to follow others, 66 students choose to plan to listen to others again, and 50 students choose to consult with others to make decisions. Regarding the question about how they feel about choosing to start a business, 103 students think they feel under some pressure. Regarding the question about what they would do after an entrepreneurial failure, nearly half of the subjects choose to gain experience and bide their time. However, in general, college students lack high entrepreneurial psychological quality, and psychologically, they are not well-prepared for entrepreneurship and lack boldness. Tables 2, 3 suggest that the basic quality of entrepreneurship of college students is generally not high, the entrepreneurial awareness is weak and the entrepreneurial psychological quality is poor. They lack entrepreneurial ability rather than entrepreneurial knowledge. The linear regression analysis of the question “What will you do after an entrepreneurship failure?” shows that the significance level of gain experience and bide their time is 0.0001, and the level of confusion is 0.015. Thus, most college student entrepreneurs will choose to gain experience and bide their time after the failure.

### Analysis of the Results of Questionnaire on Entrepreneurial Factors

**FIGURE 10:**
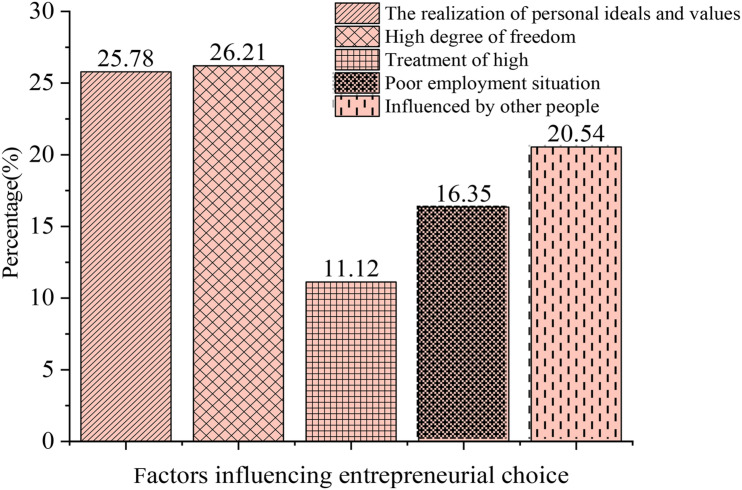
Factors influencing entrepreneurial choice.

**FIGURE 11:**
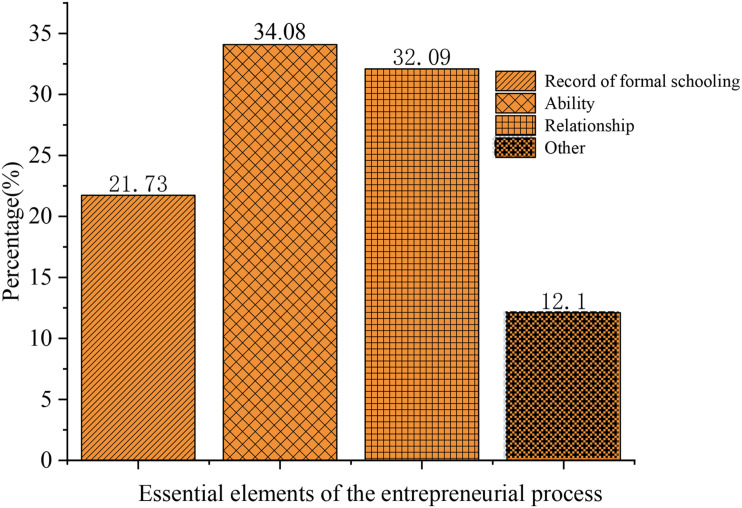
Essential elements of the entrepreneurial process.

**FIGURE 12:**
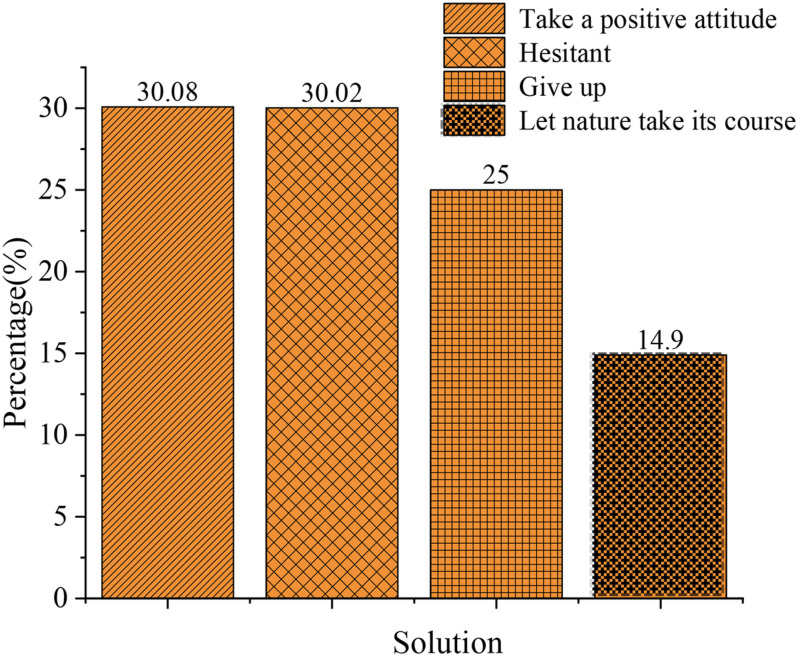
Solutions.

Figures 10–12 display the questionnaire results of entrepreneurial factors.

Figure 10 shows the questionnaire results of “What are factors influencing entrepreneurial choices?”. About 25.78% of the subjects choose to realize personal ideals and values. The answer is also in line with the cultivation values of current entrepreneurial education in higher education institutions, 26.21% of the students choose the high degree of freedom in work, and 11.12% of the students choose high treatment, indicating that they have a utilitarian tendency. About 16.35% of the subjects choose poor employment situations, and entrepreneurship is undoubtedly a new career choice. To sum up, the majority of college students think positively and healthily, and the most important factor in their entrepreneurial choices is the realization of personal ideals and values.

Figure 11 shows the questionnaire results of “What are the essential elements of the entrepreneurial process?”. About 21.65% of the subjects think that the record of formal schooling is the most important, 33.94% think that ability is the most important, and 31.97% think that relationship is the most important. The questionnaire results show that most college students think that their comprehensive quality and ability are more important during entrepreneurship, while other elements, including money, are also crucial. Entrepreneur’s ability and quality can be regarded as internal factors, and other factors are external factors. Both external factors and internal factors are essential for successful entrepreneurship.

Figure 12 shows that for the question of “What are your solutions to entrepreneurship problems?”, 30.35% of students choose to face the problem positively, 30.29% of them are hesitant, 25.23% of them choose to give up, and 14.13% of them choose to let nature take its course. Overall data imply that college students have few social experiences and low anti-risk ability. Nearly 70% of the subjects have not shown a positive attitude toward risk. Accordingly, higher education institutions should focus on values guidance in entrepreneurship education to cultivate a sound entrepreneurial psychological quality.

## Discussion on Cultivating Entrepreneurial Talents

Given the above problems, several solutions have been provided here. (1) A complete and mature external environment should be established for entrepreneurship education. (2) The entrepreneurship curriculum in higher education institutions should be improved. (3) Entrepreneurship teachers in higher education institutions should be upgraded and supplemented. (4) Practice base should be established for entrepreneurship education. In recent years, many preferential policies have been introduced to support college students’ entrepreneurship, and various training courses have also been launched. However, these measures have only been well-implemented in economically developed areas and have not been vigorously promoted throughout the country. Therefore, more high-tech industrialization bases should be established in college-concentrated areas, and the scientific and technological industries and scientific and technological activities of colleges should be promoted. In this way (Liu and Chen, 2021), the industrialization bases of colleges will gradually become the cradle of entrepreneurs. Besides, college students’ entrepreneurial projects should be assessed from multi-dimensions, and college students should be regularly trained with entrepreneurship through lectures. Meanwhile, start-up funds should be provided, and laws and regulations should be perfected, providing legal aid and protection for college students. Based on the above analysis, the entrepreneurship curriculum in higher education institutions can be improved from two aspects. First, a theoretical course on entrepreneurship should be developed. Second, practical courses and entrepreneurial activities should be increased to improve college students’ entrepreneurial quality. Entrepreneurship education is highly practical, so theory and practice should be combined. Highly experienced teachers with entrepreneurial backgrounds should be invited for a professional entrepreneurship course (Zhong et al., 2020). Practice bases can be established by higher education institutions through cooperation with high-tech enterprises, such as science-entrepreneurship park and entrepreneurship education incubation base. These practice bases can guide students to start and operate enterprises and engage in business activities, technological inventions, and services. An evaluation system should be constructed for talent cultivation: evaluation indices should be chosen first, including students’ mastery of knowledge and skills (Feng and Chen, 2020), problem-solving ability, emotions, and attitudes. Then, the evaluation standard should be determined from two aspects, including the training process and results. Finally, evaluation subjects should be diversified through the inclusion of people from all walks of life, such as students, parents, and teachers. Consequently, the evaluation results can promote the best development of students.

Method comparison: the results obtained here are compared with other recent survey methods. Thus, the advantages are as follows. The commercialization concept is introduced into the research of virtual entrepreneurship education in higher education institutions, and the existing virtual entrepreneurship education resources are integrated with Internet technology. Meanwhile, the cultivation of entrepreneurial talents integrated with the commercialization concept is explored, showing that the proposed virtual entrepreneurship education under the commercialization concept is more effective at solving problems, such as site limitation and equipment shortage, and can improve the quality of entrepreneurial talents training (Chen, 2019).

## Conclusion

The proper training mode for entrepreneurial talents is key to cultivating college students’ innovation and entrepreneurial spirit and creative ability. Higher education institutions should strengthen entrepreneurship education to cultivate students’ entrepreneurial talents. Entrepreneurship education should involve both classroom education and extracurricular practice. Based on domestic and foreign research on entrepreneurship education, the basic elements of entrepreneurial quality are summarized, and the quality index system is obtained for entrepreneurial talents. Then, the ability and quality of the existing college student entrepreneurs are tested through the quality index system during their entrepreneurship, and some strategic suggestions are put forward for the future development of entrepreneurship education in Chinese higher education institutions.

The deficiencies can be summarized as follows; there may be some deviations in sample selection in terms of gender, age, and location, and the sample coverage is not comprehensive enough. The design of the questionnaire can be further refined and optimized. It is hoped that these deficiencies will be further improved in the follow-up research.

## Data Availability Statement

The raw data supporting the conclusions of this article will be made available by the authors, without undue reservation.

## Ethics Statement

The studies involving human participants were reviewed and approved by Hangzhou Normal University Ethics Committee. The patients/participants provided their written informed consent to participate in this study. Written informed consent was obtained from the individual(s) for the publication of any potentially identifiable images or data included in this article.

## Author Contributions

Both authors listed have made a substantial, direct and intellectual contribution to the work, and approved it for publication.

## Conflict of Interest

The authors declare that the research was conducted in the absence of any commercial or financial relationships that could be construed as a potential conflict of interest.

## Publisher’s Note

All claims expressed in this article are solely those of the authors and do not necessarily represent those of their affiliated organizations, or those of the publisher, the editors and the reviewers. Any product that may be evaluated in this article, or claim that may be made by its manufacturer, is not guaranteed or endorsed by the publisher.
